# Supplementary material for: A chemical proteomics approach for global mapping of functional lysines on cell surface of living cell
Source: Nat Commun. 2024 Apr 8;15:2997. doi: 10.1038/s41467-024-47033-w (PMC11001985; doi:10.1038/s41467-024-47033-w)
Supplement: Supplementary file 1 — Supplementary Information [file 41467_2024_47033_MOESM1_ESM.pdf]

Supporting Information

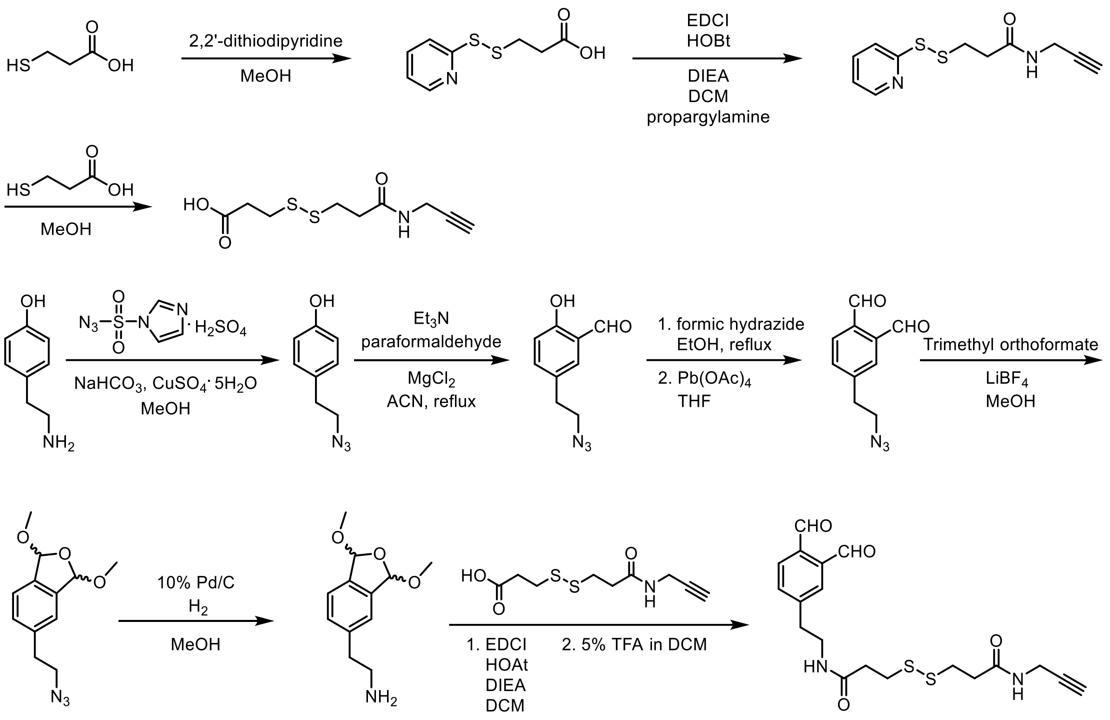

Supplementary Figure 1. Synthetic route of OPA-S-S-alkyne.

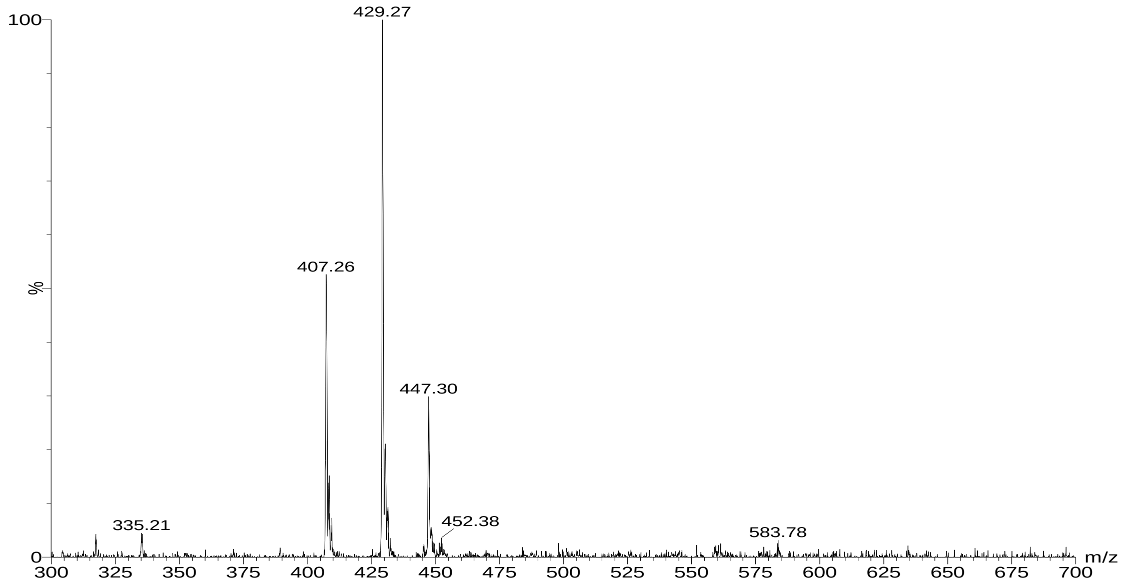

Supplementary Figure 2. ESI-MS of OPA-S-S-alkyne.

a

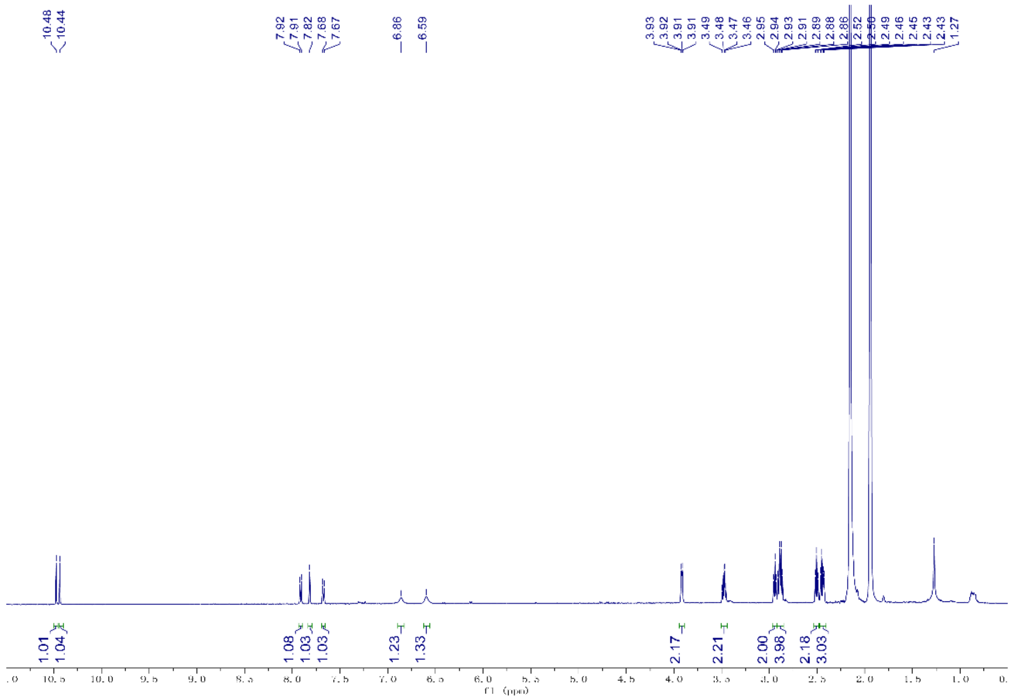

b

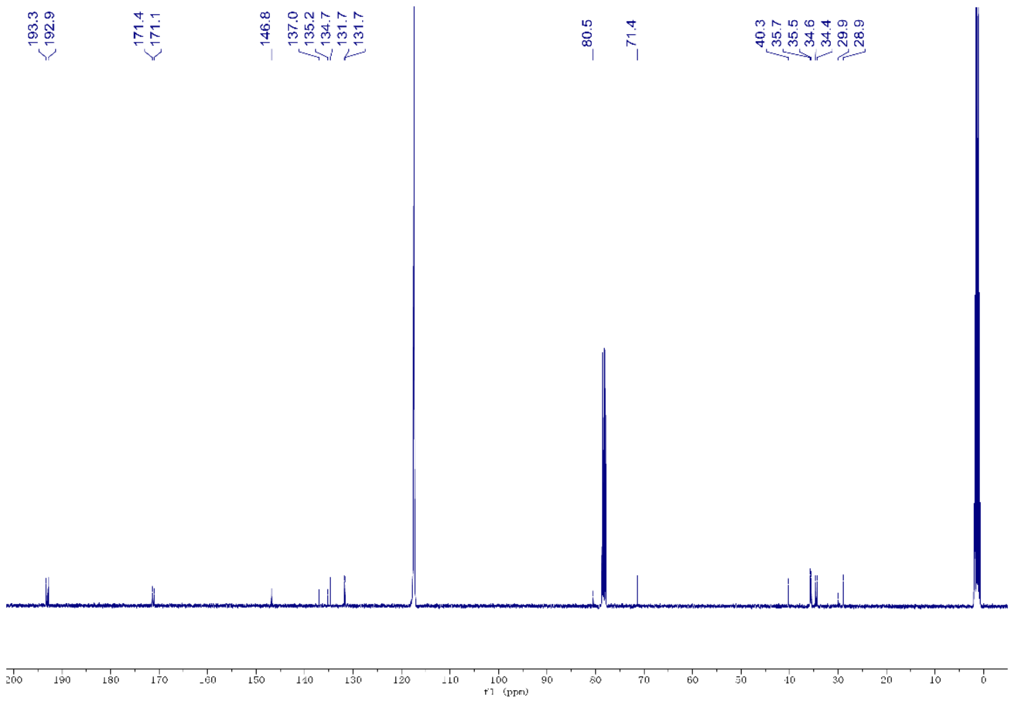

Supplementary Figure 3. (a) ^{1}H NMR spectrum (500 MHz) and (b) ^{13}C NMR spectrum (100 MHz) of OPA-S-S-alkyne in acetonitrile-d_3.

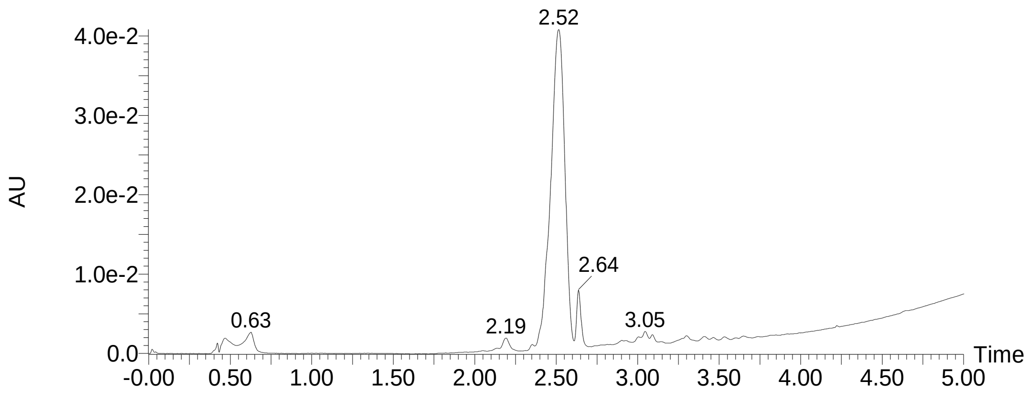

Supplementary Figure 4. RP-HPLC of OPA-S-S-alkyne probe.

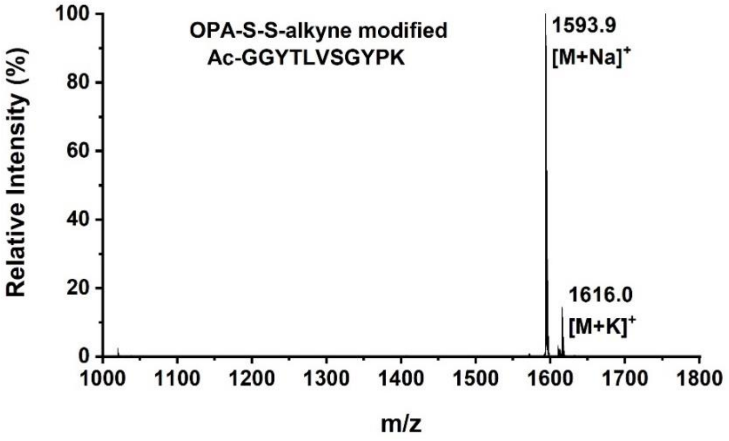

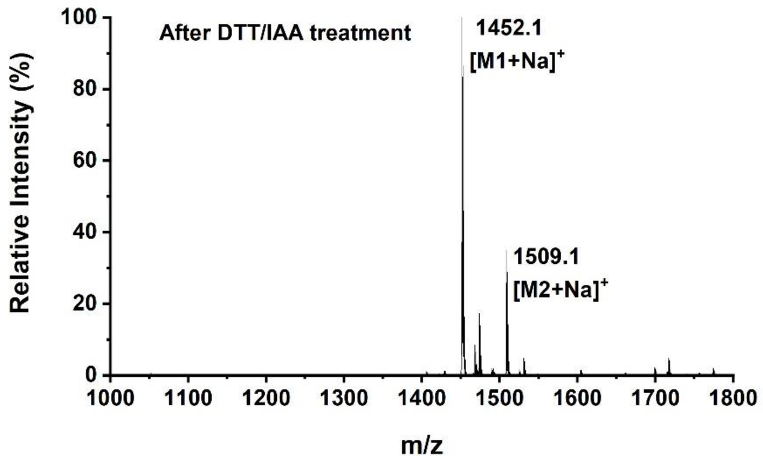

Supplementary Figure 5. Cleavage of OPA-S-S-alkyne in reducing environment. MALDI MS spectra of the OPA-S-S-alkyne modified peptide before (a) and after (b) DTT/IAA treatment. M1: the product after DTT treatment, M2: M1 derivatized by IAA.

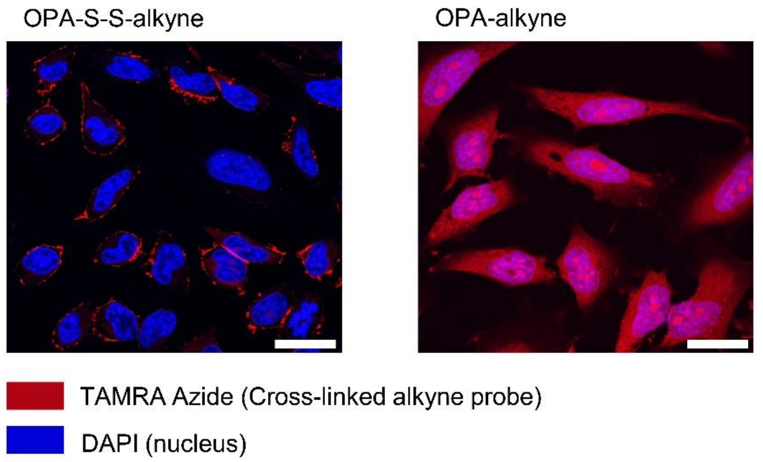

Supplementary Figure 6. Cells were labeled by OPA-S-S-alkyne (left) and OPA-alkyne (right), followed by fixation, permeabilization, click with TAMRA Azide and analyzed by confocal fluorescent microscopy. Scale bars, 25 μm.

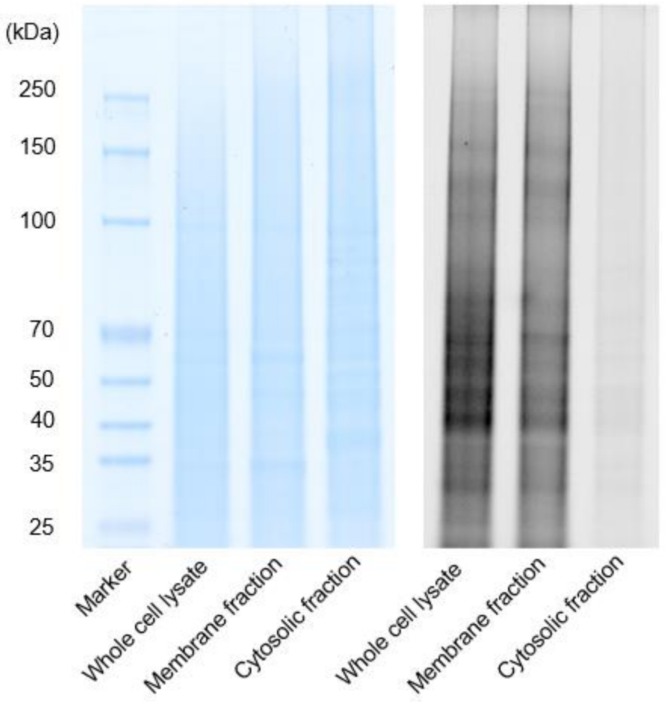

Supplementary Figure 7. Non-fractionated proteins (whole cell lysate), proteins from membrane and cytosolic fractions extracted from OPA-S-S-alkyne labeled Hela cells were clicked with TAMRA Azide and analyzed by SDS–PAGE and in-gel fluorescence scanning (right). Gels were colored by Coommassie Brilliant Blue (left) to confirm presence of all proteins in the fractions. Source data are provided as a Source Data file.

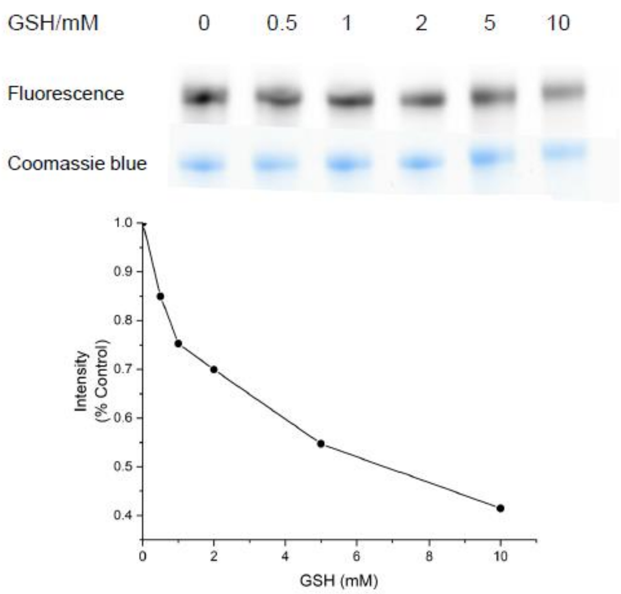

Supplementary Figure 8. OPA-S-S-alkyne labeled BSA was mixed with different concentrations of GSH, showing concentration-dependent decrease in fluorescence intensity. The gels were stained by Coomassie brilliant blue to show equal loading. Source data are provided as a Source Data file.

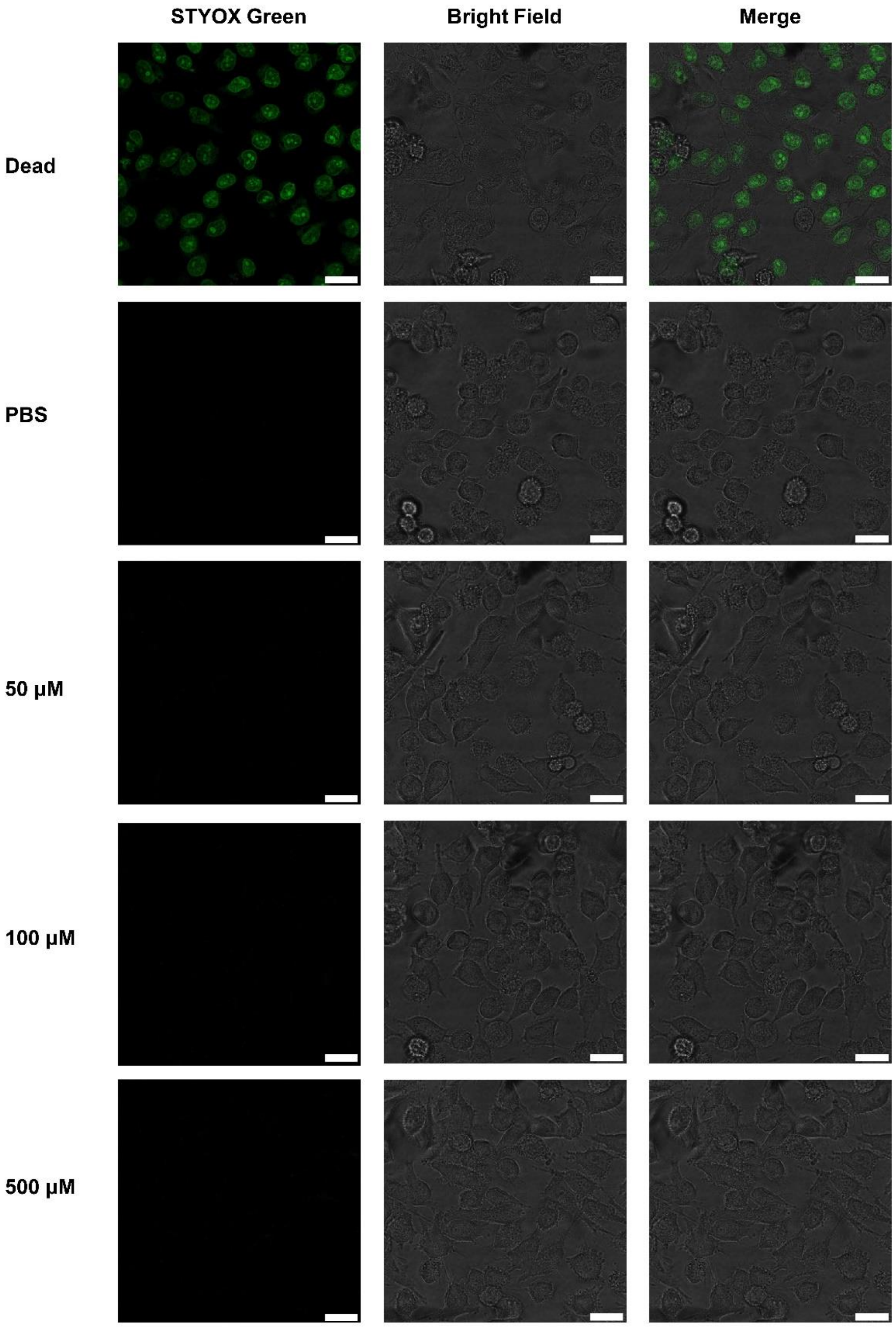

Supplementary Figure 9. Cells were treated with cold methanol or PBS or different concentrations of OPA-S-S-alkyne for 10 min and incubated with STYOX Green for 30 min, followed by confocal fluorescent microscopy analysis. Images shown are representative of three independent experiments. Scale bars, 25 μm.

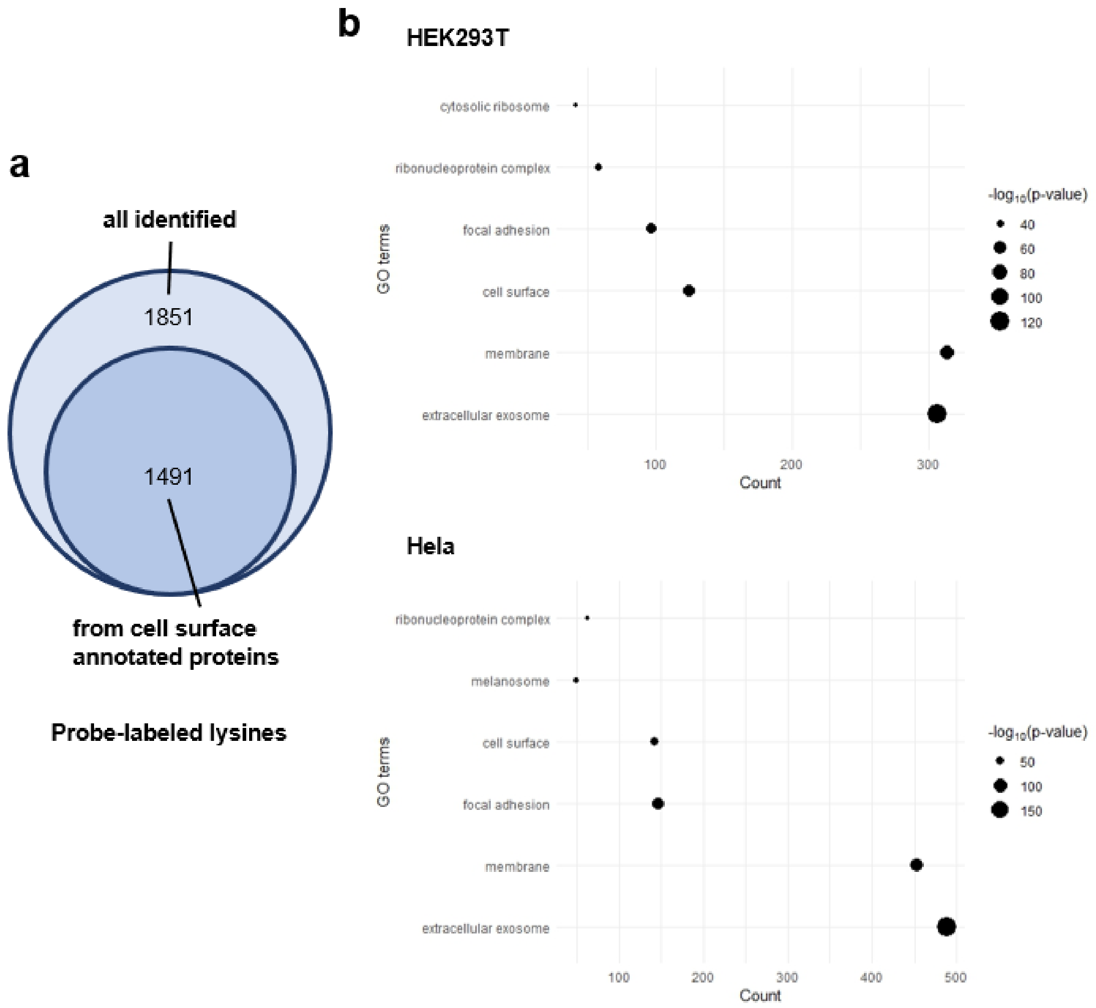

Supplementary Figure 10. a) Living HEK293T cells were labeled with OPA-S-S-alkyne (100 µM) followed by protein extraction, reacted with azide-biotin, digested by trypsin, enriched by avidin beads, eluted by DTT/IAA and analyzed by LC-MS/MS. A total of 1851 probe-labeled lysines were identified, among them 1494 lysines were from cell surface annotated proteins. b) GOCC analysis of the labeled proteins in HEK293T cells (top) and Hela cells (bottom).

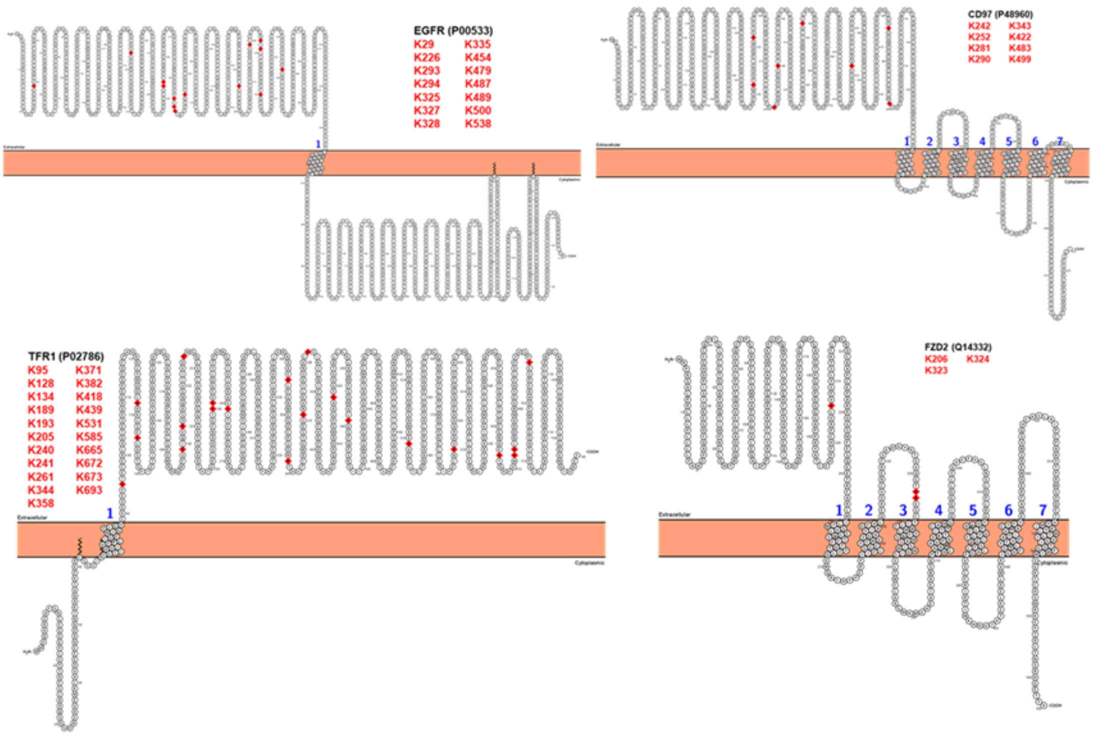

Supplementary Figure 11. Distribution of OPA-S-S-alkyne labeled lysines (red rhombuses) of four typical TMPs. EGFR: TMP with single transmembrane domain (TMD); TFR1: TMP with single TMD; CD97: TMP with 7 TMDs; FZD2: TMP with 7 TMDs. The figure was drawn using Protter[1].

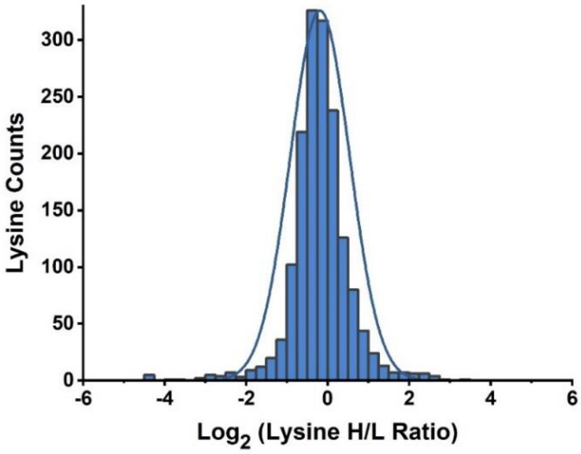

Supplementary Figure 12. Global distribution of lysine light/heavy (H/L) ratios of OPA-S-S-alkyne labeled lysines from Hela cells using GASF strategy with the heavy and light cells were both treated with 500 μM OPA-S-S-alkyne and mixed at a 1:1 ratio. The lysine heavy/light ratios had a mean of 1.01. The solid curve indicates the Gaussian fit.

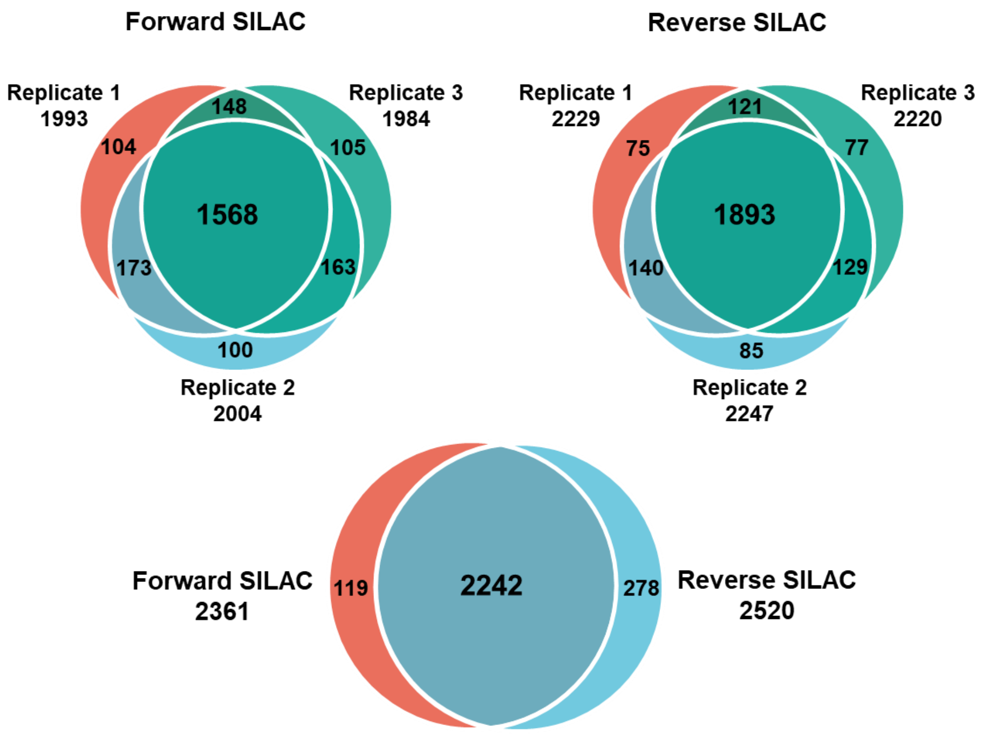

Supplementary Figure 13. Overlap of quantified OPA-S-S-alkyne labeled cell surface lysines in three replicated forward SILAC experiments (left), in three replicated reverse SILAC experiments (medium) and between the forward and reverse SILAC experiments (right).

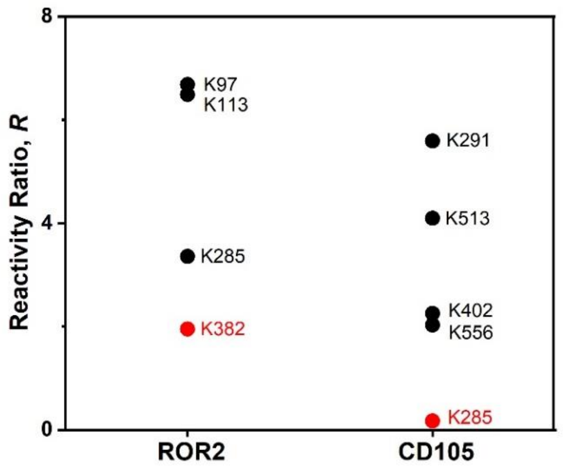

Supplementary Figure 14. *R* values for hyper-reactive (red) and medium/low-reactivity (black) lysines found within the same protein. For each protein, only a single hyper-reactive lysine was found (*R* values < 2.0) among several quantified lysines.

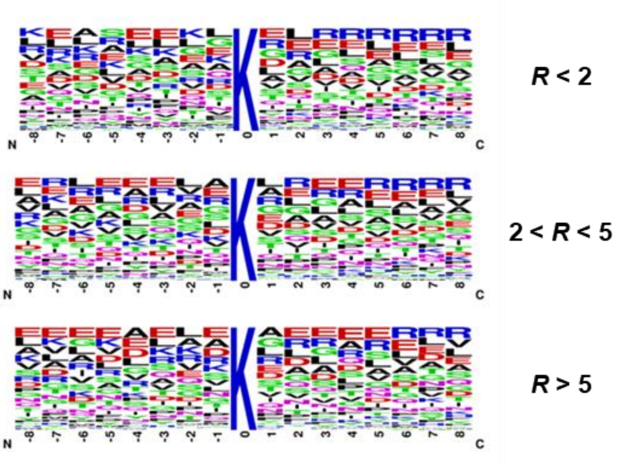

Supplementary Figure 15. Sequence motif analysis of lysines from different reactivity groups.

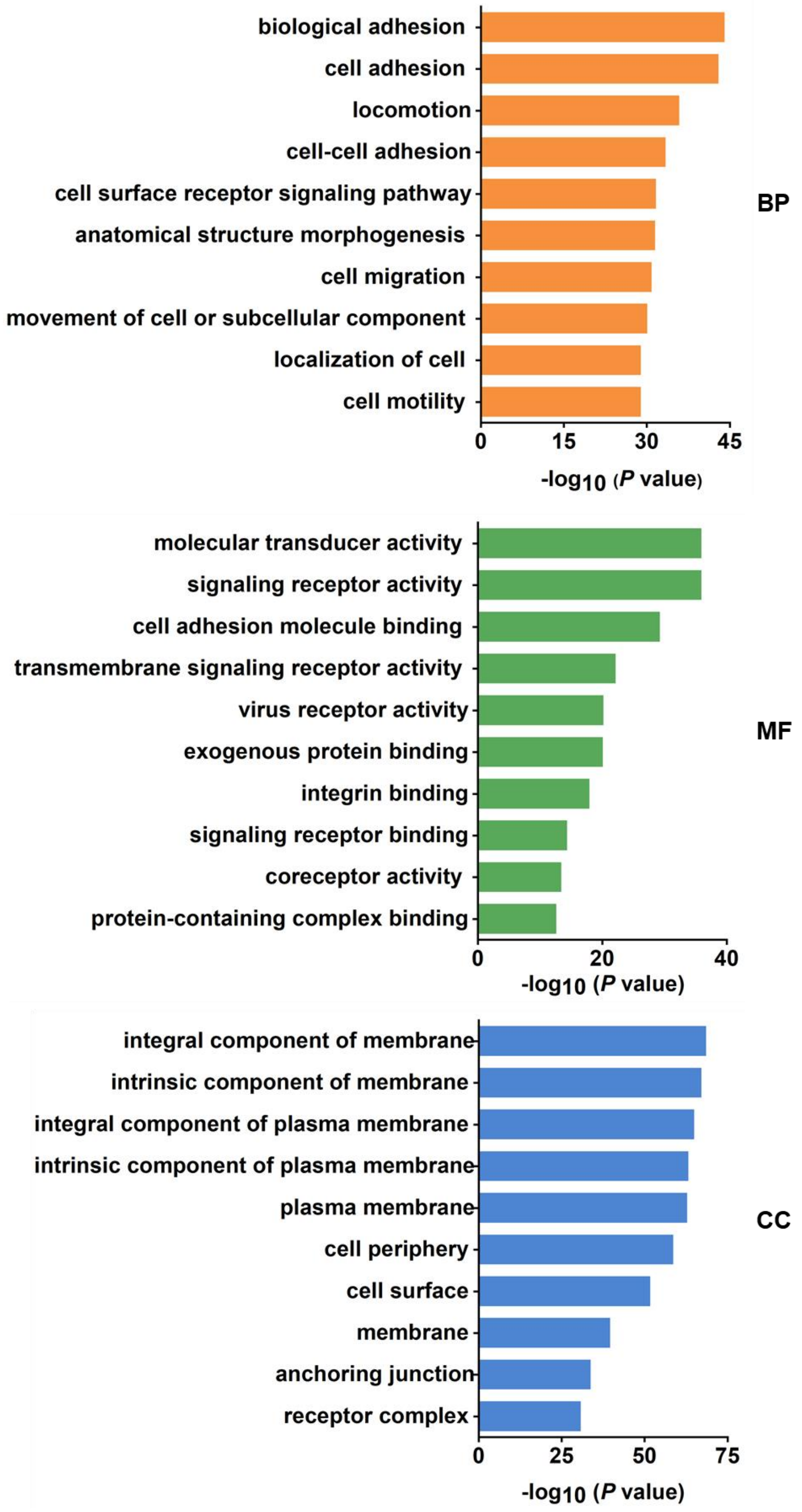

Supplementary Figure 16. Gene ontology analysis of TMPs containing hyper-reactive lysines in terms of biological process (BP, orange), molecular function (MF, green) and cellular components (CC, blue).

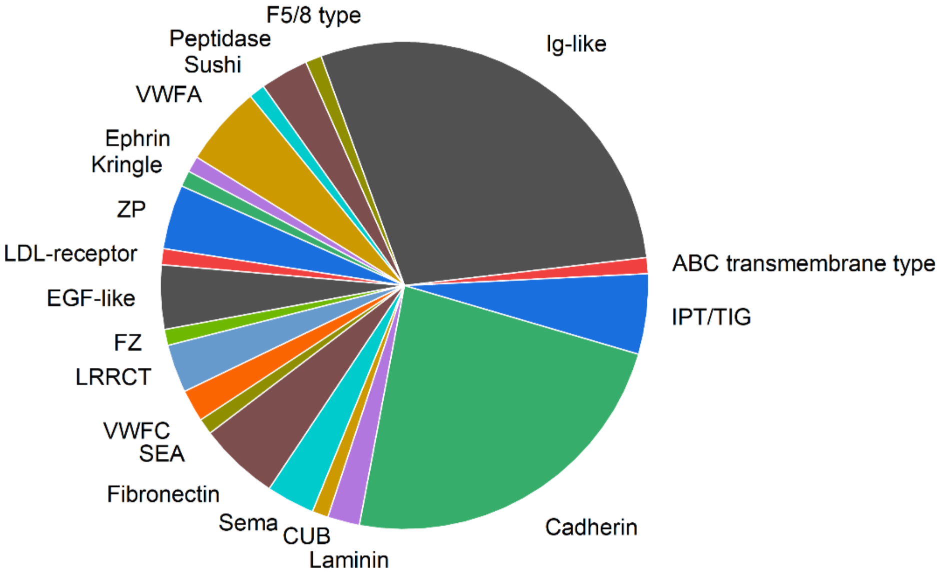

Supplementary Figure 17. Hyper-reactive lysines distribute to various protein domains.

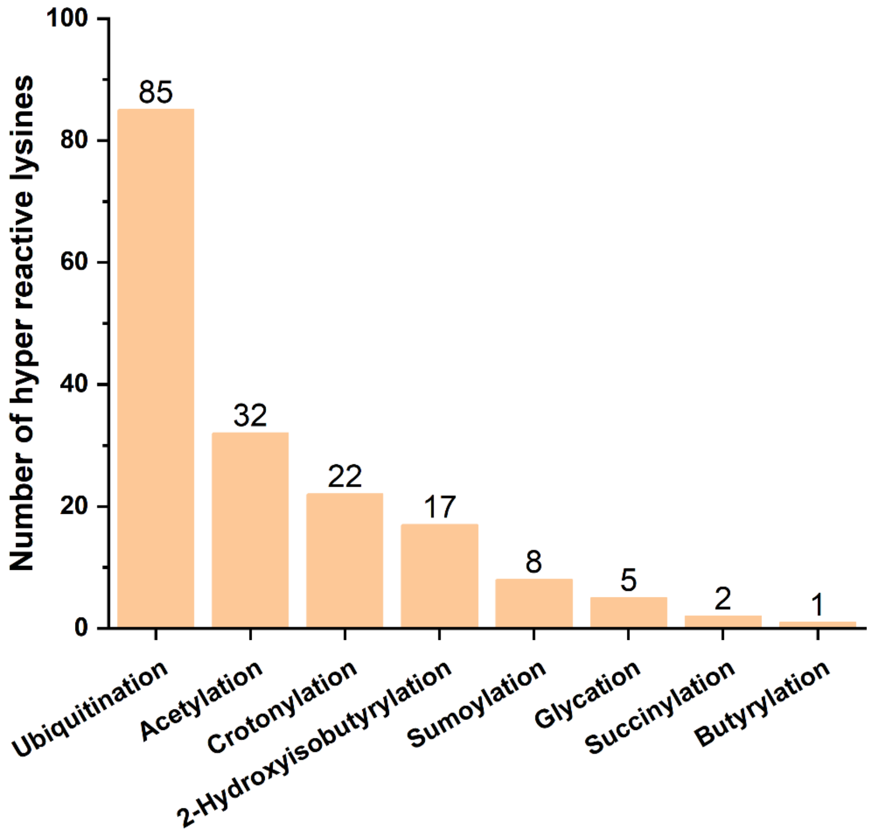

Supplementary Figure 18. Number of hyper-reactive lysines annotated to the PTMs.

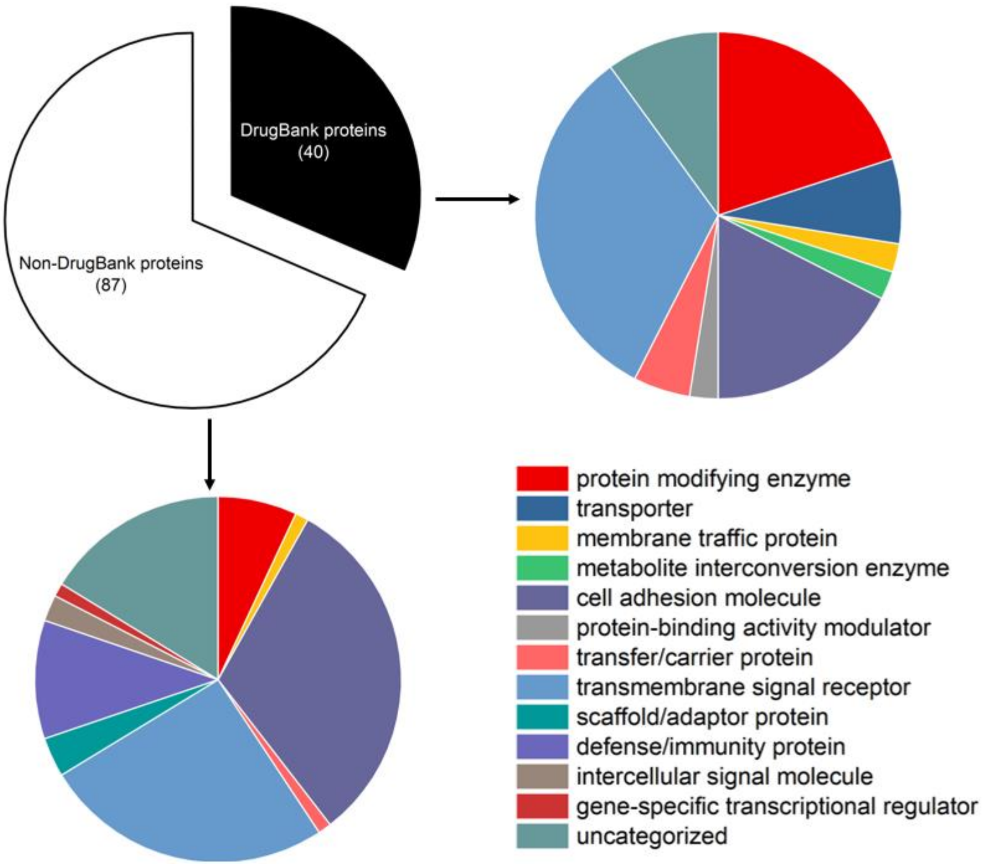

Supplementary Figure 19. Fraction of transmembrane proteins containing hyper-reactive lysines found in DrugBank and the protein class of DrugBank proteins (right) and non-DrugBank proteins(bottom).

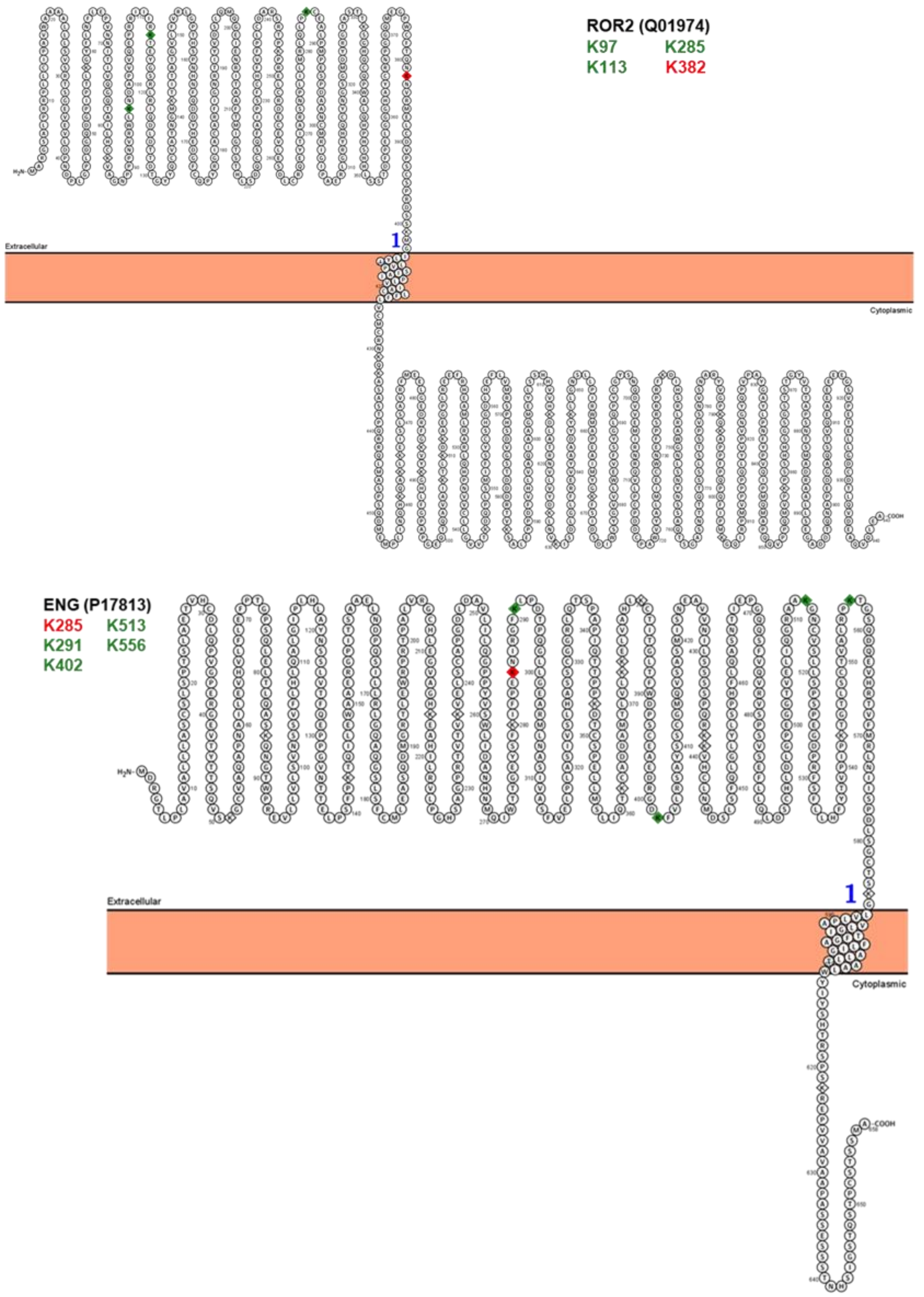

Supplementary Figure 20. Distribution of lysines of ROR2 (top) and ENG (bottom). All lysines marked with rhombuses, and quantified hyper-reactivity lysines by GASF strategy marked with red rhombuses, medium and low reactivity lysines marked with green rhombuses. The figure was drawn using Protter[1].

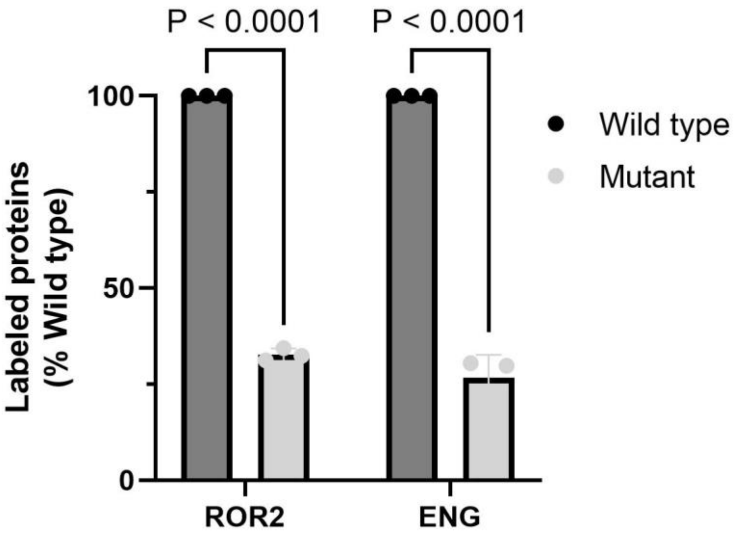

Supplementary Figure 21. Both ROR2 and ENG were labeled less by OPA-S-S-alkyne when the hyper-reactive lysine were mutated to arginine. P values (n=3, two-tailed Student's t test) and quantitative data with mean ± SD are shown in the figure. Source data are provided as a Source Data file.

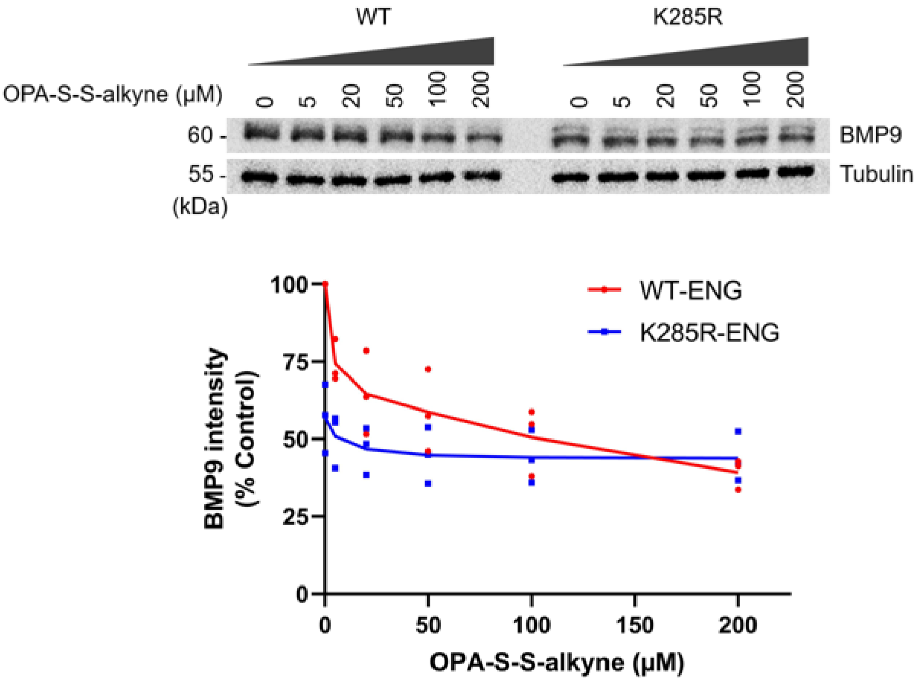

Supplementary Figure 22. Cells overexpressing WT-ENG and K285R-ENG were treated with different concentrations of OPA-S-S-alkyne *in vivo* and then lysed for western blot analysis. Quantification of western blotting data with mean from three replicates(n=3) was shown. Source data are provided as a Source Data file.

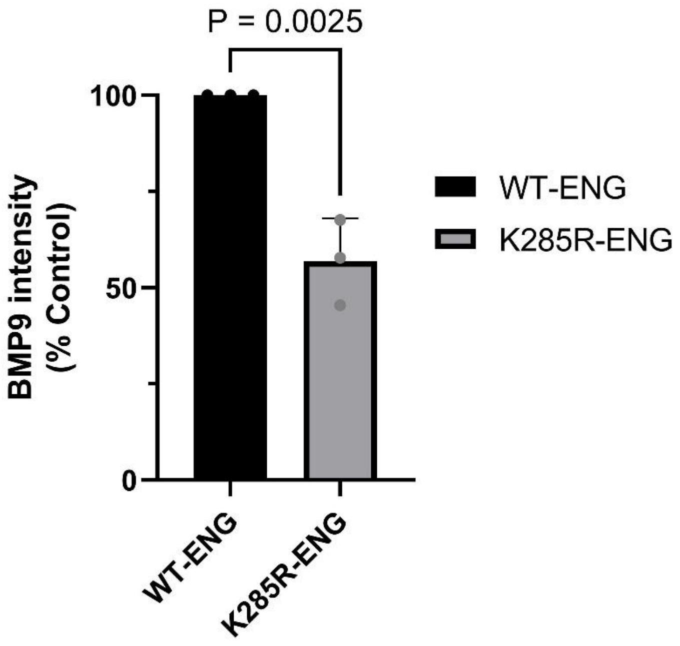

Supplementary Figure 23. The intensity of BMP9 in K285-ENG was lower than the WT-ENG without the treatment of OPA-S-S-alkyne. P value (n=3, two-tailed Student's t test) and quantitative data with mean ± SD are shown in the figure. Source data are provided as a Source Data file.

1. Omasits, U., Ahrens, C.H., Müller, S. & Wollscheid, B. Protter: interactive protein feature visualization and integration with experimental proteomic data. *Bioinformatics (Oxford, England)* **30**, 884-886 (2014).
