# Supplementary material for: A chemical proteomics approach for global mapping of functional lysines on cell surface of living cell
Source: Nat Commun. 2024 Apr 8;15:2997. doi: 10.1038/s41467-024-47033-w (PMC11001985; doi:10.1038/s41467-024-47033-w)
Supplement: Supplementary file 3 — Description of Additional Supplementary Files [file 41467_2024_47033_MOESM3_ESM.pdf]

Supplementary Data 1:

Average Reactivity Ratios across all replicate experiments for all quantified cell surface lysines that meet the quality filters described in the Methods section. Shown are R10:1 ratios for 2639 cell surface lysines.

Supplementary Data 2:

Average Reactivity Ratios across all replicate experiments for all quantified extracellular lysines of transmembrane proteins (TMPs). Shown are R10:1 ratios for 656 extracellular lysines corresponded to 197 TMPs.

Supplementary Data 3:

Average Reactivity Ratios across all replicate experiments for hyper-reactive lysines (R10:1≤2) of transmembrane proteins (TMPs). Shown are R10:1 ratios for 224 hyper-reactive lysines of TMPs as well as the designation of the functional class of the corresponding protein.
